# MicroRNA-1297 inhibits metastasis and epithelial-mesenchymal transition by targeting AEG-1 in cervical cancer

**DOI:** 10.3892/or.2022.8372

**Published:** 2022-07-19

**Authors:** Zengyan Wang, Shanyang He, Peng Guo, Xu Guo, Jingxuan Zheng

Oncol Rep 38: 3121–3129, 2017; DOI: 10.3892/or.2017.5979

Following the publication of this paper, it was drawn to the Editors' attention by a concerned reader that certain of the immunohistochemical data shown in Fig. 4C and the cell migration assay data shown in Fig. 3B and D and Fig. 7B and D were strikingly similar to data that had appeared in different form in other articles by different authors. Owing to the fact that the contentious data in the above article had already been published elsewhere, or were already under consideration for publication, prior to its submission to *Oncology Reports*, the Editor has decided that this paper should be retracted from the Journal. After having been in contact with the authors, they agreed with the decision to retract the paper. The Editor apologizes to the readership for any inconvenience caused.

